# Exercise-Induced ST-Segment Elevation in Lead aVR as a Predictor of LCx Stenosis

**Published:** 2011-12-01

**Authors:** M A Ostovan, A A Zolghadrasli

**Affiliations:** 1 Department of Cardiology, School of Medicine,Shiraz University of Medical Sciences, Shiraz, Iran; 2 Student Research Center, Shiraz University of Medical Sciences, Shiraz, Iran

**Keywords:** ST-segment elevation, aVR lead, Exercise tolerance test, Coronary angiography

Dear Editor,

Exercise-induced ST-segment depression, although the most common accepted criterion for detecting coronary artery disease (CAD) has limited sensitivity and is unable to localize ischemic territory.[1][2][3][4] Recently ST-segment elevation in lead aVR has been studied for the detection of CAD.[1][2][3][4][5][6][7] This ECG finding is usually associated with ST-segment depression in other leads, so the question is whether this ECG finding during exercise tolerance test (ETT) adds any further diagnostic information over the presence of STsegment depression alone or not? Different studies have reported exercise-induced ST-segment elevation in lead aVR to be associated with proximal left anterior descending artery (LAD), left main coronary artery (LMCA), left circumflex artery (LCx) or even right coronary artery (RCA) stenosis in various clinical settings.[1][2][3][4][5][6][7][8][9] Considering these inconclusive data, we decided to perform a cross-sectional study on patients who had positive ETT associated with ST segment elevation in lead aVR.

A total of 560 patients with symptoms resembling angina were referred to ETT room of two outpatient clinics from January 2007 through March 2009. Fifty patients with positive ETT who had co-existing ST segment elevation in lead aVR constituted the first group of study. Demographic data as well as CAD risk factors including history of hypertension, diabetes, previous history of CAD, smoking, hyperlipidemia, BMI and family history of premature CAD were obtained. Another 50 patients with positive ETT but without ST segment elevation in lead aVR who were matched one by one with the first group of patients regarding demographic data and CAD risk factors, constituted the second group of study. The study was approved by Ethical Committee of Shiraz University of Medical Sciences. P values of 0.05 or less were considered significant.

Regarding the number of vessels involved (i.e. single, two or three vessel disease), there was no statistical significance (Chi-Square test, p value = 0.740), but regarding the type of coronary vessel, it was shown that LCx was involved significantly higher in the first group than the second group (Chi- Square test, p value = 0.016, Table 1). This finding was in accordance with the finding of Gaitonde et al. that significant proximal LCx stenosis should cause ST-segment elevation in both lead aVR and V1, reflecting posterior wall ischemia. Using vectorcardiology, they proposed ECG changes in the posterolateral wall to be reflected in lead aVR.[10] Our study provided further evidences in support of this hypothesis although not supported by other investigators.

This study was unique in the way that it was designed in the form of a cross-sectional study only focusing on ST-segment elevation in lead aVR. So, further studies are needed to confirm this finding. It was also demonstrated that exercise induced ST-segment elevation in lead aVR had a sensitivity of 64%, specificity of 50%, positive predictive value of 32% and negative predictive value of 21% in predicting LCx stenosis. These numbers may not be comparable with the results reported in other studies, but considering the fact that the stenosis of LCx was one of the main causes of false negative results in ETT, its importance may become more apparent.

Some studies have concentrated on specific groups of patients, either during or shortly after myocardial infarction while our selection of study population was performed on a large group of patients with a broad spectrum of demographic data and therefore more applicable to actual clinical setting. One of the limitations of our study was the low number of patients.

Another limitation is that the sensitivity, specificity, negative and positive predictive value provided were calculated among patients with positive ETT and so can not be extended to the whole population.

**Table 1:** Angiographic findings of the patients

**Vessel involvement**	**ST-segment elevation in lead aVR (Group 1) (%) **	**No ST-segment elevation in lead aVR (Group 2) (%) **	***P* value**
LMCA	-	2(4.0)	0.495
LAD	20(48)	22(44)	0.841
LCx	30(60)	17(34)	0.016
RCA	21(42)	20(40)	0.673
Normal angiography	18(36)	18(36)	
